# Elucidation of key lactylation- and palmitoylation-related genes and their related mechanisms in the development of neuropathic pain

**DOI:** 10.1097/MD.0000000000050651

**Published:** 2026-10-02

**Authors:** Honglin Yan, Lei Li, Jing Li, Junqiang Hao, Yanwei Yang

**Affiliations:** a Department of Anesthesiology, Chuiyangliu Hospital Affiliated with Tsinghua University, Beijing, China.

**Keywords:** biomarkers, immune infiltration, lactylation, neuropathic pain, NOD-like receptor signaling, palmitoylation

## Abstract

Neuropathic pain (NP) is a debilitating disorder with heterogeneous etiologies and limited reliable biomarkers for diagnosis and therapeutic targeting. This study aimed to identify key lactylation- and palmitoylation-related genes involved in NP and to elucidate their potential regulatory mechanisms. NP-related transcriptomic datasets (GSE124272, GSE150408, and GSE95849) were retrieved from the Gene Expression Omnibus (GEO). Differentially expressed genes associated with lactylation and palmitoylation were identified and analyzed using functional enrichment, correlation analysis, and protein–protein interaction networks. Feature genes were selected using support vector machine–recursive feature elimination and XGBoost algorithms. Diagnostic performance was assessed using receiver operating characteristic (ROC) curve analysis, and a predictive nomogram was constructed. Immune infiltration was evaluated by single-sample gene set enrichment analysis, while gene set enrichment analysis, miRNA–mRNA, and transcription factor–mRNA network analyses were performed to explore regulatory mechanisms. Independent clinical validation was conducted by quantitative real-time PCR using peripheral blood samples from NP patients (n = 17) and matched healthy controls (n = 17). Hematopoietic cell kinase (HCK; lactylation-related) and WASP actin nucleation-promoting factor (WAS; palmitoylation-related) were identified as NP feature genes. Both genes were significantly upregulated in NP samples across training and validation GEO datasets and were independently confirmed in the clinical cohort. HCK and WAS demonstrated diagnostic utility (area under the curve > 0.7 in GEO analyses), and a nomogram based on these genes showed favorable calibration and decision-curve benefit. Expression of both genes was positively correlated with infiltration of neutrophils, monocytes, and macrophages and was enriched in the NOD-like receptor signaling pathway. Regulatory network analysis revealed interactions between HCK and miRNAs including hsa-miR-21-5p and hsa-miR-146a-5p, and between WAS and miRNAs including hsa-miR-34a-5p and hsa-miR-16-5p. Both genes were predicted to be regulated by the transcription factor SPI1. WAS and HCK represent promising peripheral blood biomarkers for NP and may contribute to NP pathogenesis through immune cell–mediated neuroinflammation and NOD-like receptor signaling, highlighting potential targets for future diagnostic and therapeutic development.

## 1. Introduction

Neuropathic pain (NP) is a chronic and refractory pain syndrome characterized by spontaneous pain, hyperalgesia, allodynia, and sensory abnormalities.^[1]^ It results from lesions or diseases affecting the somatosensory nervous system and impacts approximately 10% of the global population.^[2]^ Current clinical interventions often fail to provide adequate symptom relief for many NP patients. Commonly used analgesics, including opioids and nonsteroidal anti-inflammatory drugs, frequently provide insufficient efficacy and are associated with adverse effects.^[3]^ Persistent pain, together with impaired mental health and social functioning, markedly diminishes quality of life and creates a substantial socioeconomic burden.^[4]^ Despite clinical prevalence and impact, NP mechanisms remain incompletely defined, and reliable biomarkers that can support diagnosis, stratification, and therapeutic monitoring are still limited. Posttranslational modifications (PTMs) increasingly appear central to neuroimmune interactions and pain sensitization. Lactylation is a recently identified PTM implicated in neurodevelopment and neurological disorders.^[5]^ Histone lactylation, including H3K18 lactylation, can modulate gene transcription through chromatin remodeling.^[6]^ Lactylation is not restricted to histones; nonhistone proteins such as PKM2 may undergo lactylation with functional consequences for enzyme activity and immunometabolism.^[7]^ Emerging evidence links lactylation-associated signaling to innate immune pathways, including regulation of cGAS–STING signaling and type I interferon responses.^[8,9]^ Because type I interferon signaling can modulate neuroinflammation and pain sensitivity, lactylation-related regulatory programs may be relevant to NP pathophysiology, but systematic biomarker identification remains limited. Palmitoylation is another PTM that modulates membrane association, trafficking, and signaling of numerous proteins and is catalyzed by DHHC palmitoyltransferases.^[10]^ Palmitoylation influences neuronal excitability and glial activation, both of which are key drivers of pain hypersensitivity. For example, hyperpalmitoylation of glial fibrillary acidic protein can promote astrocyte proliferation and neurodegenerative pathology.^[11]^ Glial fibrillary acidic protein palmitoylation in spinal astrocytes has been reported to contribute to NP development.^[12]^ In addition, palmitoylation of A-kinase anchoring protein 150 facilitates synaptic incorporation of AMPA receptors and enhances pain hypersensitivity.^[13]^ However, comprehensive identification of palmitoylation-related biomarkers in NP remains insufficient.

Here, we integrated multiple publicly available transcriptomic datasets to identify lactylation- and palmitoylation-related genes associated with NP. We combined differential expression, network analysis, and machine learning to nominate feature genes and build a diagnostic nomogram. We further explored immune infiltration patterns and regulatory networks involving miRNAs and transcription factors (TFs). Importantly, we performed independent clinical validation using quantitative real-time polymerase chain reaction (qRT-PCR) in peripheral blood to improve translational robustness.

## 2. Materials and methods

### 2.1. Ethics statement

The clinical validation study was approved by the institutional ethics committee of Chuiyangliu Hospital Affiliated with Tsinghua University (Approval No. CHTU/66/55/24). Written informed consent was obtained from all participants. The Gene Expression Omnibus (GEO) datasets used in this study are publicly available and were analyzed in accordance with relevant guidelines.

### 2.2. Study design overview

This study consisted of a discovery phase using GEO datasets (training: merged GSE124272 + GSE150408; validation: GSE95849), computational validation of diagnostic performance (receiver operating characteristic [ROC] and nomogram), mechanistic inference using immune infiltration, gene set enrichment analysis (GSEA), and regulatory networks, and independent clinical validation using qRT-PCR in peripheral blood (NP n = 17; controls n = 17).

### 2.3. Data acquisition and preprocessing

Two NP-related gene expression datasets, GSE124272 (8 NP samples and 8 normal controls)^[14]^ and GSE150408 (7 NP samples and 17 normal controls),^[15]^ were obtained from GEO using the GEOquery package (version 2.66.0).^[16]^ Both datasets were generated on the GPL21185 platform. Raw probe matrices and platform annotations were downloaded; probes were mapped to gene symbols, probes without valid mapping were removed, and genes represented by multiple probes were summarized by their mean expression value.

After preprocessing, the datasets were merged. Principal component analysis (PCA) was performed using FactoMineR to evaluate batch effects. Batch correction was conducted using ComBat (SVA package).^[17]^ PCA was repeated to verify that batch-driven variation was minimized while preserving biological group structure.

GSE95849 (6 NP samples and 6 normal controls),^[18]^ generated on GPL22448, was processed similarly and used as an independent validation dataset.

A total of 327 lactylation-related genes were obtained from Cheng et al,^[19]^ and 167 palmitoylation-related genes were obtained from the GeneCards database.

### 2.4. Differential expression analysis

Differentially expressed genes (DEGs) between NP and control samples were identified using limma (version 3.54.2).^[20]^ Thresholds were set at |log_2_ fold change| > 0.263 and *P* < .05.^[21]^ The relatively lenient fold-change cutoff was used to capture potentially meaningful transcriptional shifts in complex inflammatory conditions where effect sizes in whole-blood expression can be modest. Where appropriate, Benjamini–Hochberg adjustment was applied for enrichment analyses; raw *P* thresholds for DEG screening were retained to prevent over-filtering in small datasets, and downstream validation steps were used to reduce false positives.

Lactylation-related and palmitoylation-related DEGs were obtained by intersecting total DEGs with the respective gene sets.

### 2.5. Functional enrichment analysis

Gene Ontology (GO)^[22]^ and Kyoto Encyclopedia of Genes and Genomes (KEGG)^[23]^ enrichment analyses were performed using ClusterProfiler (version 4.6.2).^[24]^ Adjusted *P* < .05 and count ≥ 2 were considered significant.

### 2.6. Correlation analysis

Correlations between lactylation- and palmitoylation-related DEGs were calculated using the R cor.test function. Significant correlations were defined as |*R*| > 0.7 and *P* < .05.

### 2.7. Protein–protein interaction (PPI) network construction and hub gene selection

PPI interactions were retrieved from STRING^[25]^ with an interaction score ≥ 0.4 and visualized in Cytoscape (version 3.10.2).^[26]^ Hub genes were ranked using cytoHubba by maximal clique centrality; the top 10 genes were considered hub candidates.

### 2.8. Feature gene selection by machine learning

Feature selection was conducted using support vector machine–recursive feature elimination (e1071 package, version 1.7-13)^[27]^ and XGBoost (version 1.7.7.1). Candidate feature genes were defined as the intersection of genes selected by both approaches.

### 2.9. Expression validation and diagnostic performance

Candidate feature genes were validated for differential expression in both the merged training cohort and GSE95849 using limma. Diagnostic performance was evaluated with ROC analysis using pROC (version 1.18.4).^[28]^ Genes showing consistent direction of change across cohorts and area under the curve (AUC) > 0.7 were retained as final feature genes.

### 2.10. Diagnostic nomogram construction and clinical utility assessment

A nomogram based on feature genes was constructed using the merged training cohort (GSE124272 and GSE150408) and rms (version 6.7-1).^[29]^ Calibration plots were generated to assess agreement between predicted and observed probabilities. Decision curve analysis and clinical impact curves were performed using rmda (version 1.6).^[30]^

### 2.11. Immune infiltration analysis

Single-sample gene set enrichment analysis (ssGSEA) was performed using GSVA (version 1.46.0) to estimate immune cell infiltration scores from whole-blood expression profiles. Between-group differences were assessed using *t*-tests. Pearson correlation evaluated associations between feature gene expression and immune cell infiltration scores.

### 2.12. Gene–gene interaction (GGI) and GSEA

GeneMANIA was used to generate GGI networks. For GSEA, samples were stratified into high- and low-expression groups by the median expression of each feature gene. ClusterProfiler was used for GSEA, with significance defined as adjusted *P* < .05 and |NES| > 1.

### 2.13. miRNA–mRNA and TF–mRNA regulatory networks

miRNA–mRNA and TF–mRNA interactions were obtained from miRNet and visualized using Cytoscape.

### 2.14. Small-molecule drug prediction

Potential small-molecule drugs associated with feature genes were predicted using DSigDB.

### 2.15. Participants, inclusion/exclusion criteria, and sampling

An independent clinical cohort was recruited for qRT-PCR validation, comprising patients with NP (n = 17) and age- and sex-matched healthy controls (n = 17). Adults aged 20 to 70 years were eligible for inclusion in the NP group if they had a clinically confirmed diagnosis of chronic NP with a duration of ≥ 3 months. Diagnoses were established by experienced clinicians based on typical NP symptoms, neurological examination, and supportive investigations where appropriate. The NP cohort primarily included patients with sciatica and diabetic peripheral neuropathy. Participants were excluded if they had a history of autoimmune disease or systemic inflammatory disorders, acute infection within the preceding 4 weeks, malignancy, severe hepatic, renal, or cardiovascular dysfunction, other chronic pain disorders, use of immunosuppressive or immunomodulatory therapy within the preceding 3 months, pregnancy or breastfeeding, major psychiatric illness that could affect informed consent or compliance, or inadequate peripheral blood samples for RNA extraction. Healthy controls were recruited from routine health examination populations and were included if they had no history of NP, chronic pain conditions, neurological disease, or acute or chronic inflammatory disorders.

### 2.16. RNA extraction, reverse transcription, and qRT-PCR

Peripheral venous blood was collected into anticoagulant tubes and processed according to standardized procedures. Total RNA was extracted using TRIzol reagent following the manufacturer’s instructions. RNA purity and concentration were assessed (A260/A280 between 1.8 and 2.0). cDNA was synthesized using a commercial reverse transcription kit.

qRT-PCR was performed using SYBR Green chemistry on a real-time PCR system. Gene-specific primers quantified WASP actin nucleation-promoting factor (WAS) and hematopoietic cell kinase (HCK) expression. GAPDH served as the housekeeping control. Relative expression was calculated using the 2^−ΔΔCt^ method. The primers are listed in Table S1, Supplemental Digital Content 1.

### 2.17. Statistical analysis

Statistical analyses were performed using R software (version 4.3.1) and GraphPad Prism (version 9.5.1). Data distribution was evaluated using the Shapiro–Wilk test. For comparisons between 2 groups, an unpaired Student *t* test was applied to normally distributed variables, while the Mann–Whitney *U* test was used for non-normally distributed variables. For immune cell infiltration analyses derived from ssGSEA, between-group comparisons were conducted using the Wilcoxon rank-sum test. Correlations between feature gene expression and immune cell infiltration scores were assessed using Pearson correlation analysis. To account for multiple testing in immune infiltration and enrichment analyses, *P* values were adjusted using the Benjamini–Hochberg false discovery rate method where appropriate. ROC curve analysis was performed to evaluate the diagnostic performance of individual genes and combined models. AUC values with 95% confidence intervals (CIs) were calculated using DeLong method. A combined diagnostic model integrating WAS and HCK expression was constructed using binary logistic regression. All statistical tests were two-sided, and an adjusted or unadjusted *P* value < .05 was considered statistically significant, as specified for each analysis.

## 3. Results

### 3.1. Identification of lactylation- and palmitoylation-related DEGs

PCA based on the merged datasets revealed clear batch-associated variation between GSE124272 and GSE150408 (Fig. 1A). After ComBat batch correction, inter-dataset differences were substantially reduced (Fig. 1B). Subsequent PCA colored by phenotype demonstrated preserved separation between NP and control samples (Fig. 1C). DEG analysis in the merged training cohort identified 1805 DEGs between NP and controls (874 upregulated, 931 downregulated; Fig. 1D). A heatmap demonstrated overall segregation of NP and controls based on DEG expression patterns (Fig. 1E). Intersection with lactylation- and palmitoylation-related gene sets yielded 30 lactylation-related DEGs and 22 palmitoylation-related DEGs. These lactylation- and palmitoylation-related DEGs represented 1.4% and 1.1% of total DEGs, respectively (Fig. 1F–G).

**Figure 1.**
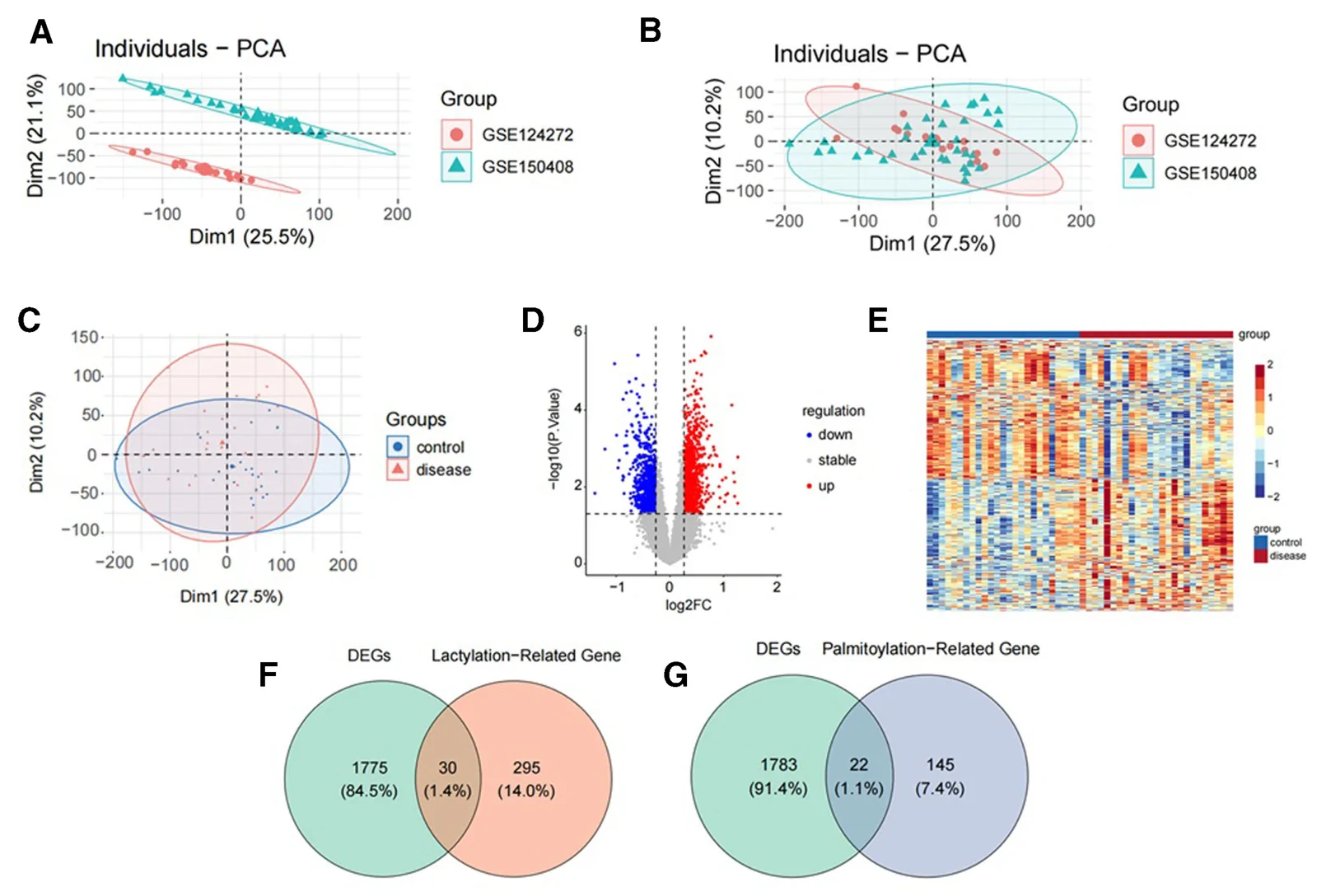
Identification of lactylation- and palmitoylation-related differentially expressed genes (DEGs). (A) Principal component analysis (PCA) of the merged GSE124272 and GSE150408 datasets prior to batch correction, colored by dataset, demonstrating clear batch-associated variation. (B) PCA after ComBat batch-effect correction, showing reduced inter-dataset separation between GSE124272 and GSE150408. (C) PCA of batch-corrected samples colored by phenotype (neuropathic pain vs control), demonstrating preservation of disease–control separation. (D) Volcano plot of DEGs between neuropathic pain (NP) and control samples (red, upregulated; blue, downregulated). (E) Heatmap illustrating expression patterns of DEGs across NP and control samples. (F) Venn diagram showing overlap between DEGs and lactylation-related genes. (G) Venn diagram showing overlap between DEGs and palmitoylation-related genes. DEGs = differentially expressed genes, PCA = principal component analysis.

### 3.2. Functional enrichment analysis of lactylation- and palmitoylation-related DEGs

Lactylation-related DEGs were enriched in multiple GO cellular component terms, including nucleoplasm, focal adhesion, and ribosomal components (Fig. 2A). No GO biological process terms met the adjusted significance threshold, likely reflecting the limited gene set size and stringent multiple-testing correction. KEGG analysis indicated enrichment in mismatch repair and DNA replication pathways (Fig. 2B), suggesting lactylation-related dysregulation may relate to transcriptional and replicative programs detectable in peripheral blood. Palmitoylation-related DEGs were enriched across GO biological process terms (e.g., cellular response to chemical stimulus), cellular component terms (e.g., vesicle), and molecular function terms (e.g., anion binding; Fig. 2C). No KEGG pathways met adjusted significance thresholds in this subset, potentially due to gene number constraints and pathway coverage.

**Figure 2.**
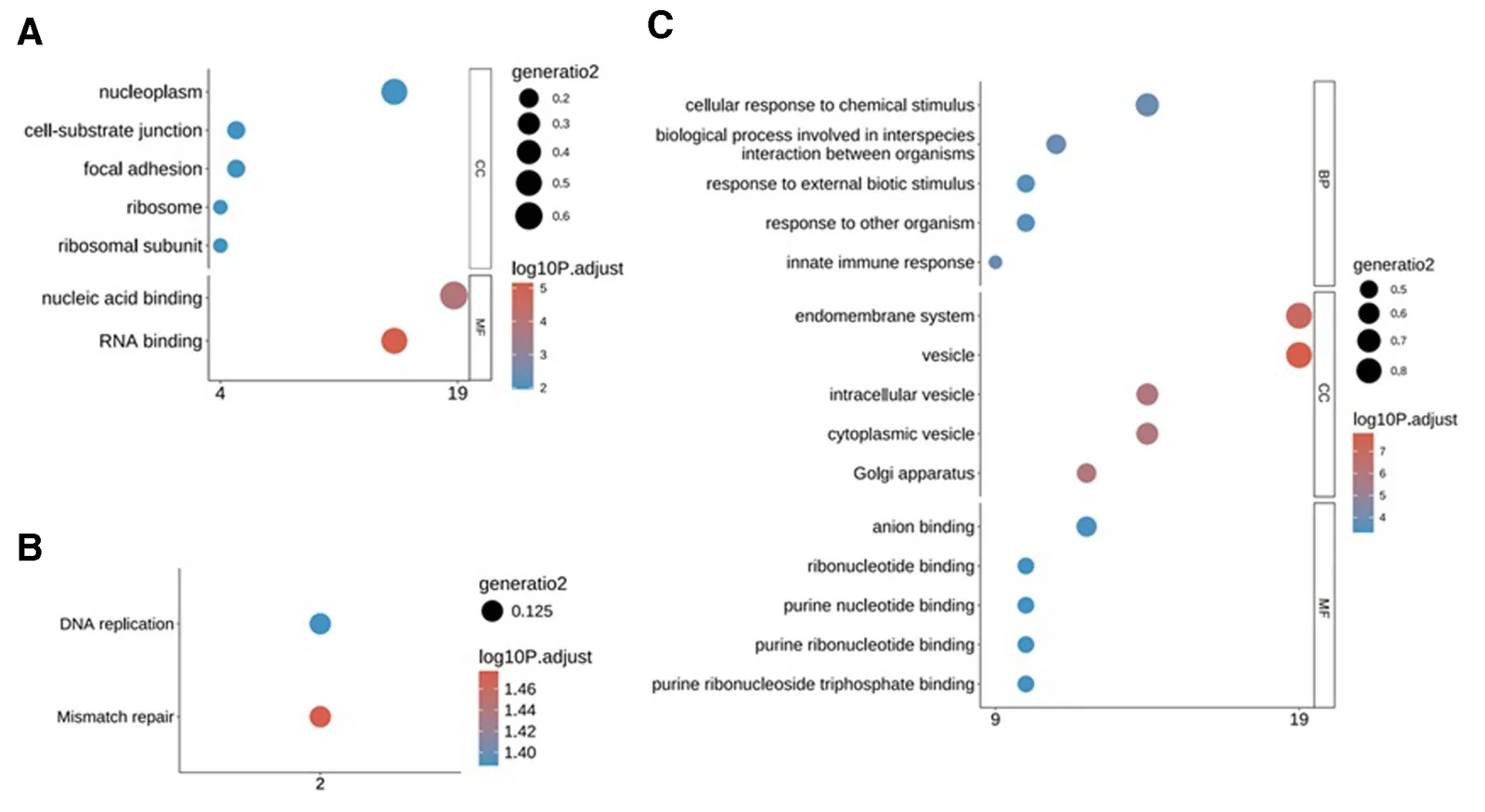
Functional enrichment analysis of lactylation- and palmitoylation-related DEGs. (A) Gene Ontology (GO) enrichment analysis of lactylation-related DEGs, highlighting enriched cellular component and molecular function categories. (B) Kyoto Encyclopedia of Genes and Genomes (KEGG) pathway enrichment analysis of lactylation-related DEGs. (C) GO enrichment analysis of palmitoylation-related DEGs across biological process, cellular component, and molecular function categories. GO = Gene Ontology, KEGG = Kyoto Encyclopedia of Genes and Genomes.

### 3.3. Correlation analysis and PPI network construction identify Hub candidates

Correlation analysis between lactylation- and palmitoylation-related DEGs identified 39 significantly correlated genes (Fig. 3A–B). STRING-based PPI analysis of these genes produced an interaction network (Fig. 3C). Maximal clique centrality ranking highlighted 10 hub candidates: RPS27A, RPL5, WAS, SRP14, FKBP3, HCK, GNAQ, RPL24, HSPE1, and NPM1.

**Figure 3.**
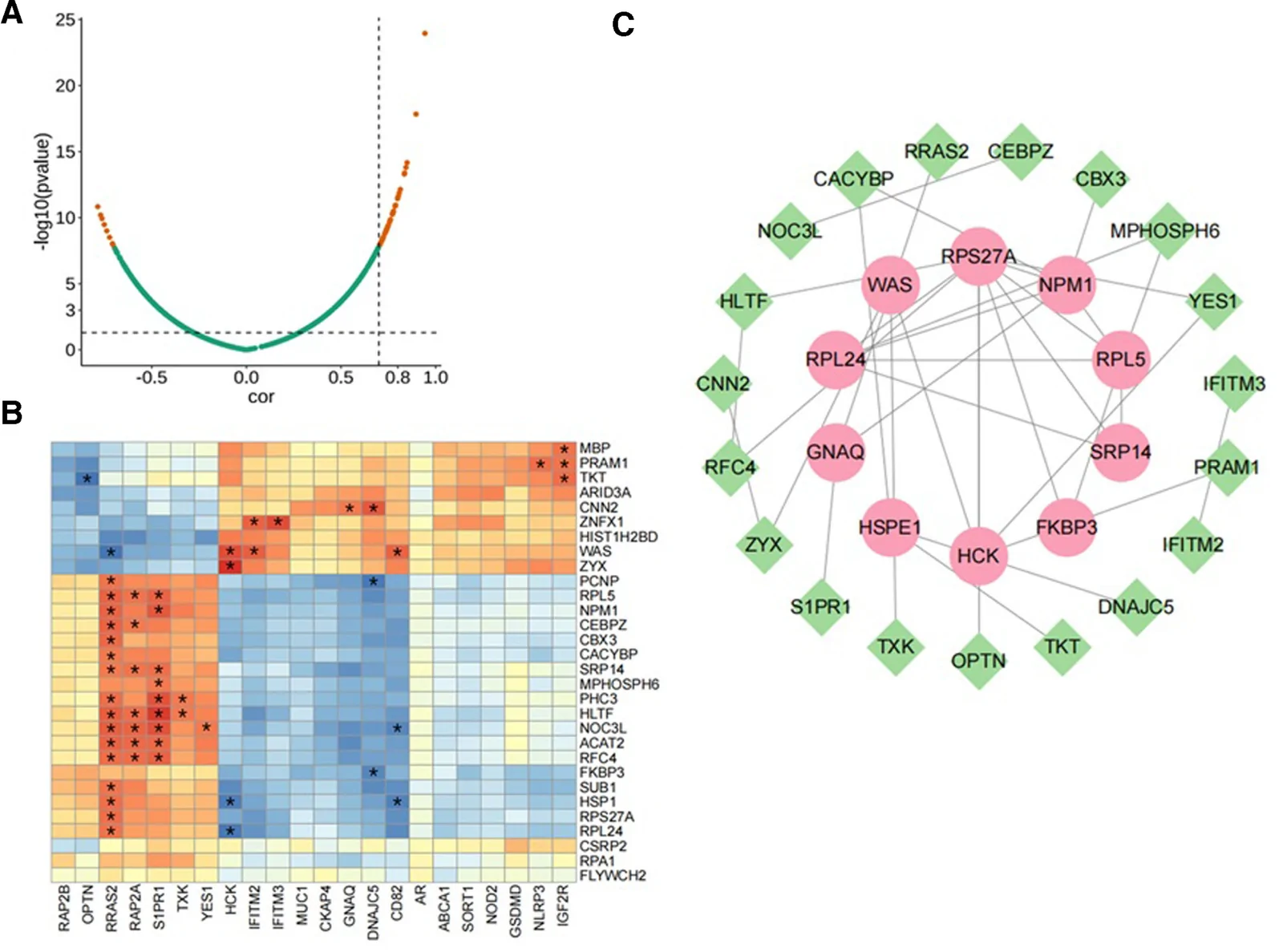
Correlation analysis and protein–protein interaction (PPI) network of lactylation- and palmitoylation-related DEGs. (A) Scatter plot showing correlations between lactylation- and palmitoylation-related DEGs. (B) Correlation heatmap of significantly correlated genes (*P* < .05). (C) PPI network constructed from correlated genes using STRING, with hub genes identified based on maximal clique centrality (MCC). DEGs = differentially expressed genes, PPI = protein–protein interaction.

### 3.4. Machine-learning feature selection identifies WAS and HCK as NP feature genes

Support vector machine–recursive feature elimination selected 7 top-performing genes with a cross-validation accuracy of 0.688 (Fig. 4A), while XGBoost ranked gene importance (Fig. 4B). Intersection yielded 7 candidate feature genes (WAS, GNAQ, RPL5, HSPE1, FKBP3, HCK, and SRP14). In both the merged training cohort and the independent GEO validation dataset (GSE95849), WAS and HCK consistently showed significant upregulation in NP (Fig. 4C). ROC analysis demonstrated diagnostic utility for WAS and HCK in both cohorts (Fig. 4D), meeting the predefined AUC threshold. The extremely high AUC values observed in GSE95849 should be interpreted with caution due to the small sample size of this dataset, which may inflate performance estimates.

**Figure 4.**
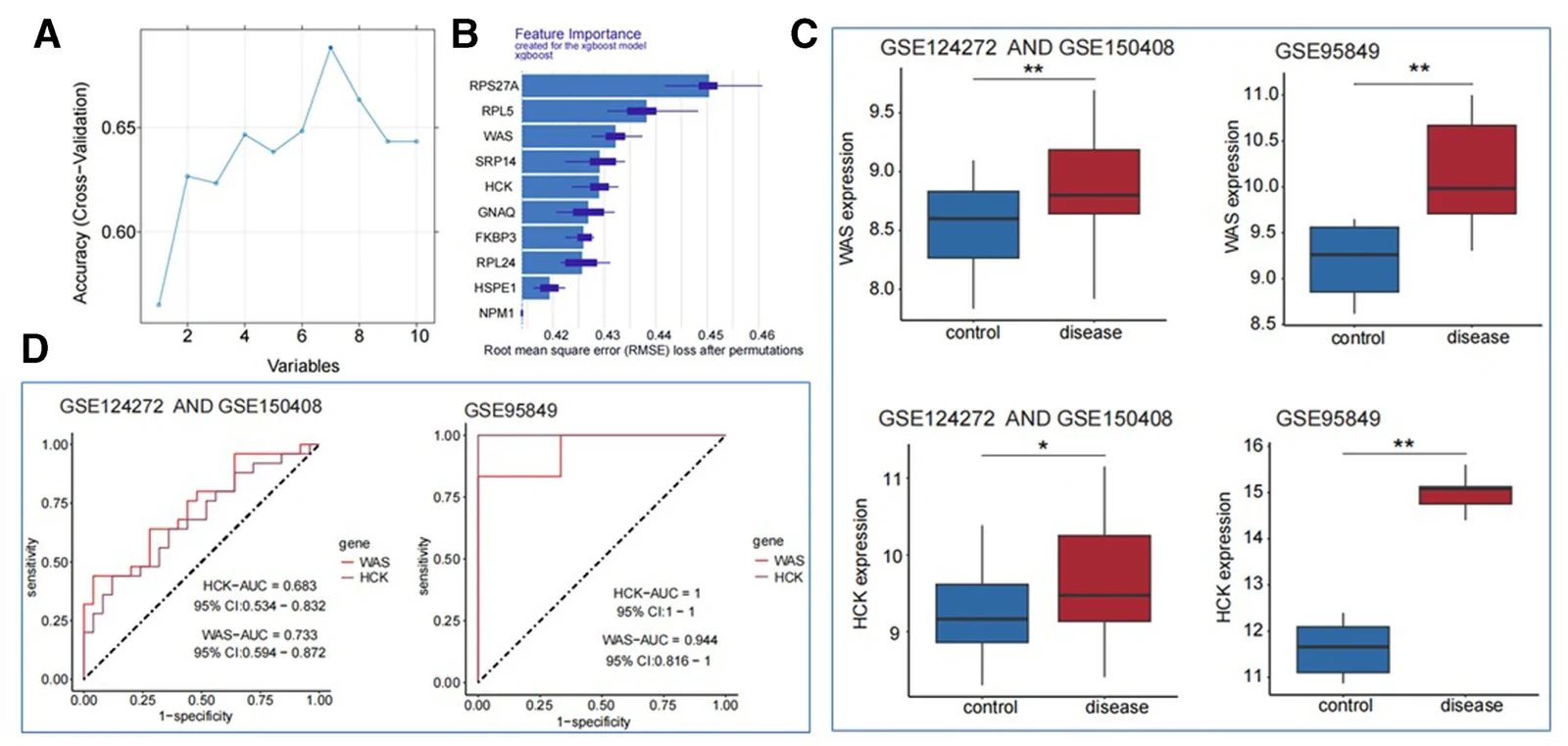
Identification and validation of feature genes. (A) Support vector machine–recursive feature elimination (SVM-RFE) showing cross-validation accuracy across increasing numbers of selected variables. (B) XGBoost feature importance ranking based on permutation-based root mean square error (RMSE) loss. (C) Expression levels of WAS and HCK in the merged training cohort (GSE124272 and GSE150408) and the independent validation dataset (GSE95849). (D) Receiver operating characteristic (ROC) curves evaluating the diagnostic performance of WAS and HCK in training and validation datasets. AUC = area under the curve, HCK = hematopoietic cell kinase, NP = neuropathic pain, ROC = receiver operating characteristic, WAS = WASP actin nucleation-promoting factor.

### 3.5. Diagnostic nomogram construction and evaluation

A two-gene nomogram incorporating WAS and HCK was constructed (Fig. 5A). Calibration analysis showed agreement between predicted and observed NP probabilities (Fig. 5B). Decision curve analysis suggested that the two-gene model provided greater net benefit across relevant threshold probabilities compared with single-gene models (Fig. 5C). Clinical impact curves further supported potential clinical utility (Fig. 5D).

**Figure 5.**
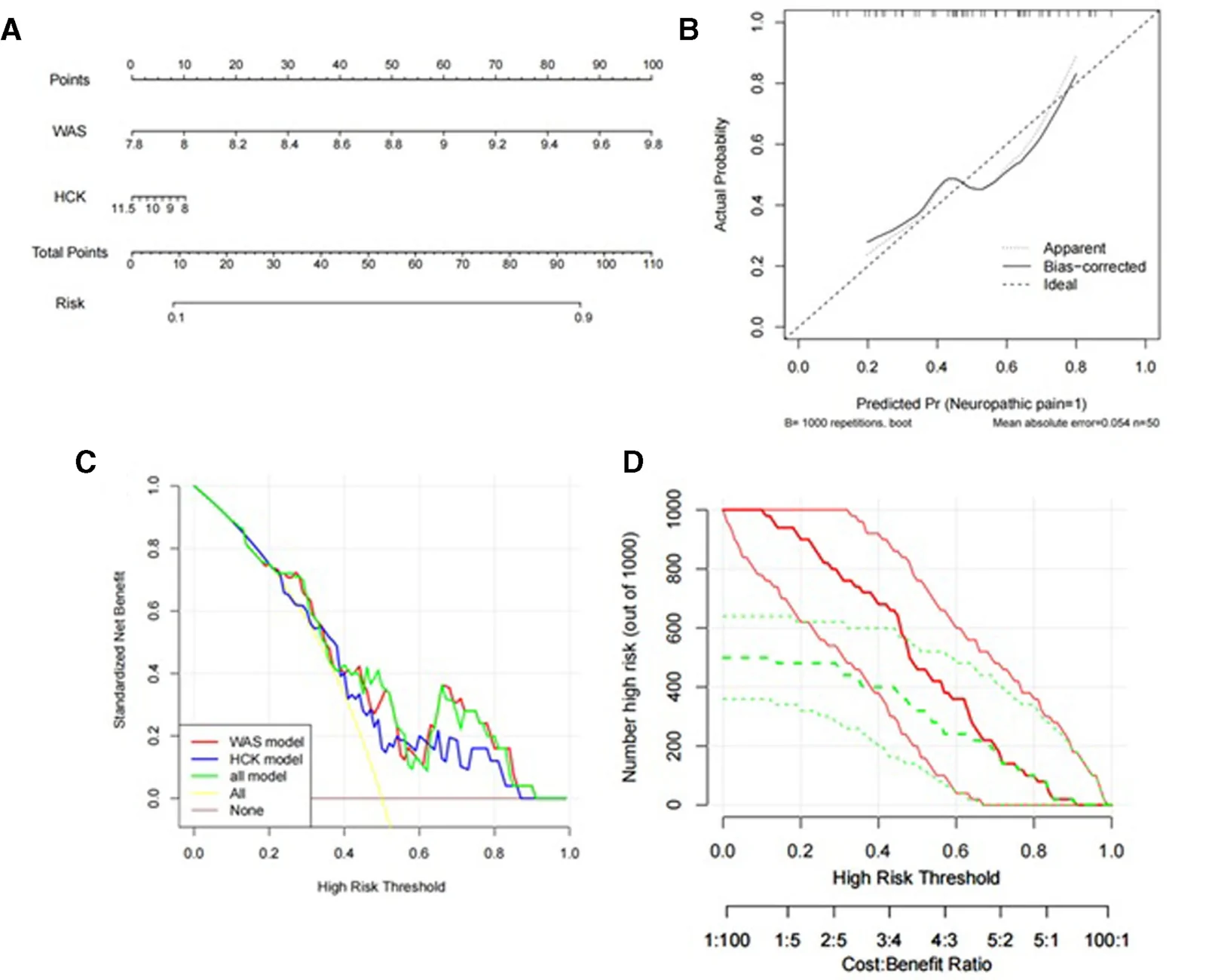
Construction and evaluation of the diagnostic nomogram based on WAS and HCK. (A) Nomogram integrating WAS and HCK expression for estimating the probability of neuropathic pain. (B) Calibration curve assessing agreement between predicted and observed probabilities. (C) Decision curve analysis (DCA) comparing the clinical net benefit of the combined model with single-gene models across a range of threshold probabilities. (D) Clinical impact curve illustrating the estimated number of high-risk individuals at different risk thresholds. DCA = decision curve analysis, HCK = hematopoietic cell kinase, NP = neuropathic pain, WAS = WASP actin nucleation-promoting factor.

### 3.6. Immune infiltration patterns and correlations with WAS and HCK

ssGSEA revealed significant between-group differences in 9 immune cell types, including mast cells, NK cells, activated CD4 T cells, myeloid-derived suppressor cells, macrophages, monocytes, neutrophils, activated dendritic cells, and activated CD8 T cells (Fig. 6A). WAS and HCK exhibited positive correlations with neutrophils, monocytes, and macrophages and negative correlations with effector memory CD4 T cells (*P* < .01; Fig. 6B–D), supporting an association with innate immune activation in NP-related peripheral blood signatures.

**Figure 6.**
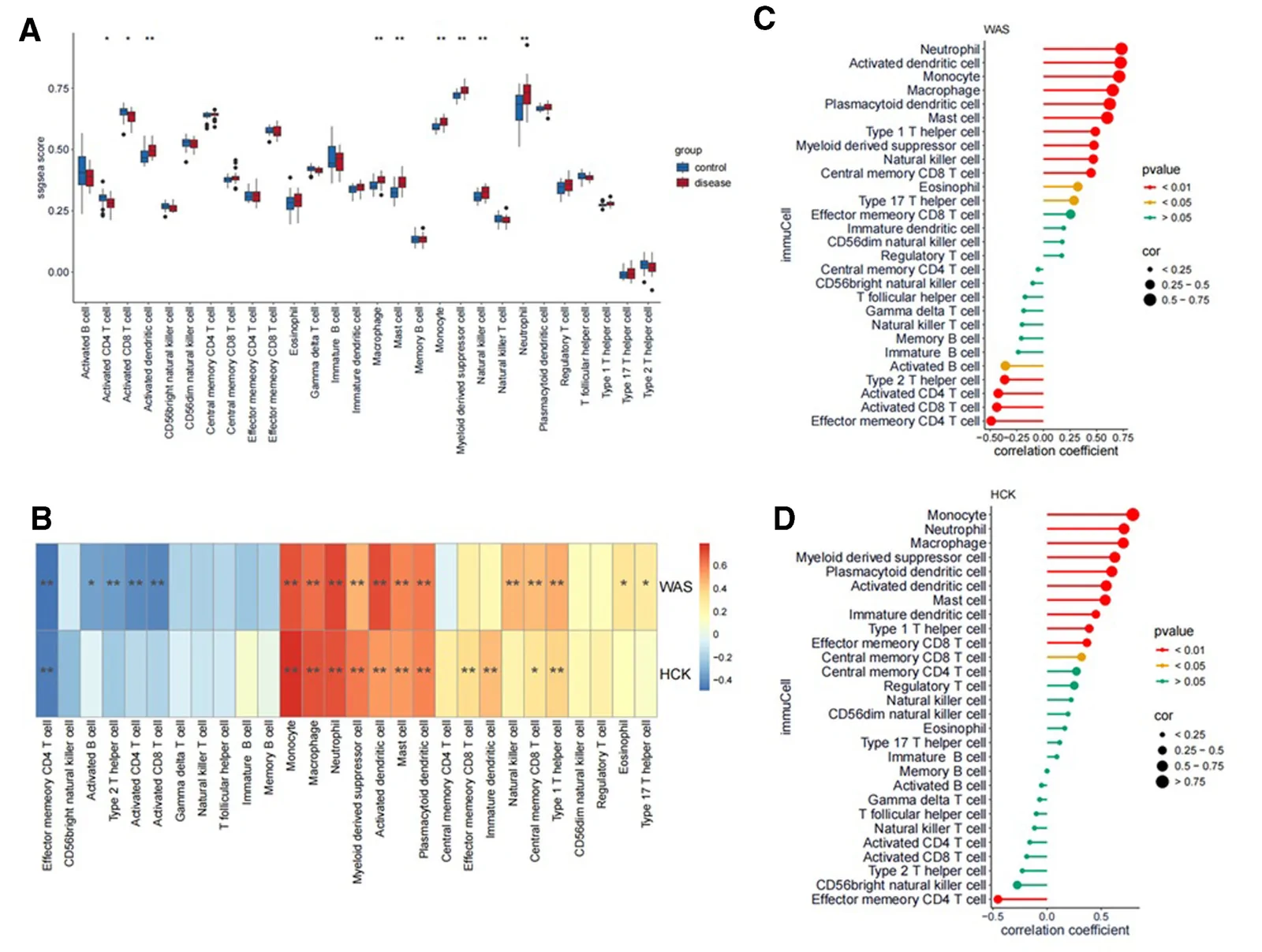
Immune infiltration patterns and correlations with WAS and HCK expression. (A) Differences in inferred infiltration levels of 28 immune cell types between NP and control samples based on single-sample gene set enrichment analysis (ssGSEA). (B) Pearson correlation heatmap showing associations between feature gene expression and immune cell infiltration scores. (C) Correlation analysis between WAS expression and immune cell infiltration levels. (D) Correlation analysis between HCK expression and immune cell infiltration levels. HCK = hematopoietic cell kinase, NP = neuropathic pain, WAS = WASP actin nucleation-promoting factor.

### 3.7. Potential regulatory mechanisms: GGI, GSEA, miRNA–mRNA, and TF–mRNA networks

GeneMANIA analysis indicated that WAS and HCK were connected with genes involved in immune signaling processes (e.g., LCP2 and WIPF1) and Fc receptor-associated pathways (Fig. 7A). GSEA revealed enrichment of immune/inflammatory programs, including upregulated NOD-like receptor signaling and downregulated ribosomal pathways in high-expression groups for both WAS and HCK (Fig. 7B–C).

**Figure 7.**
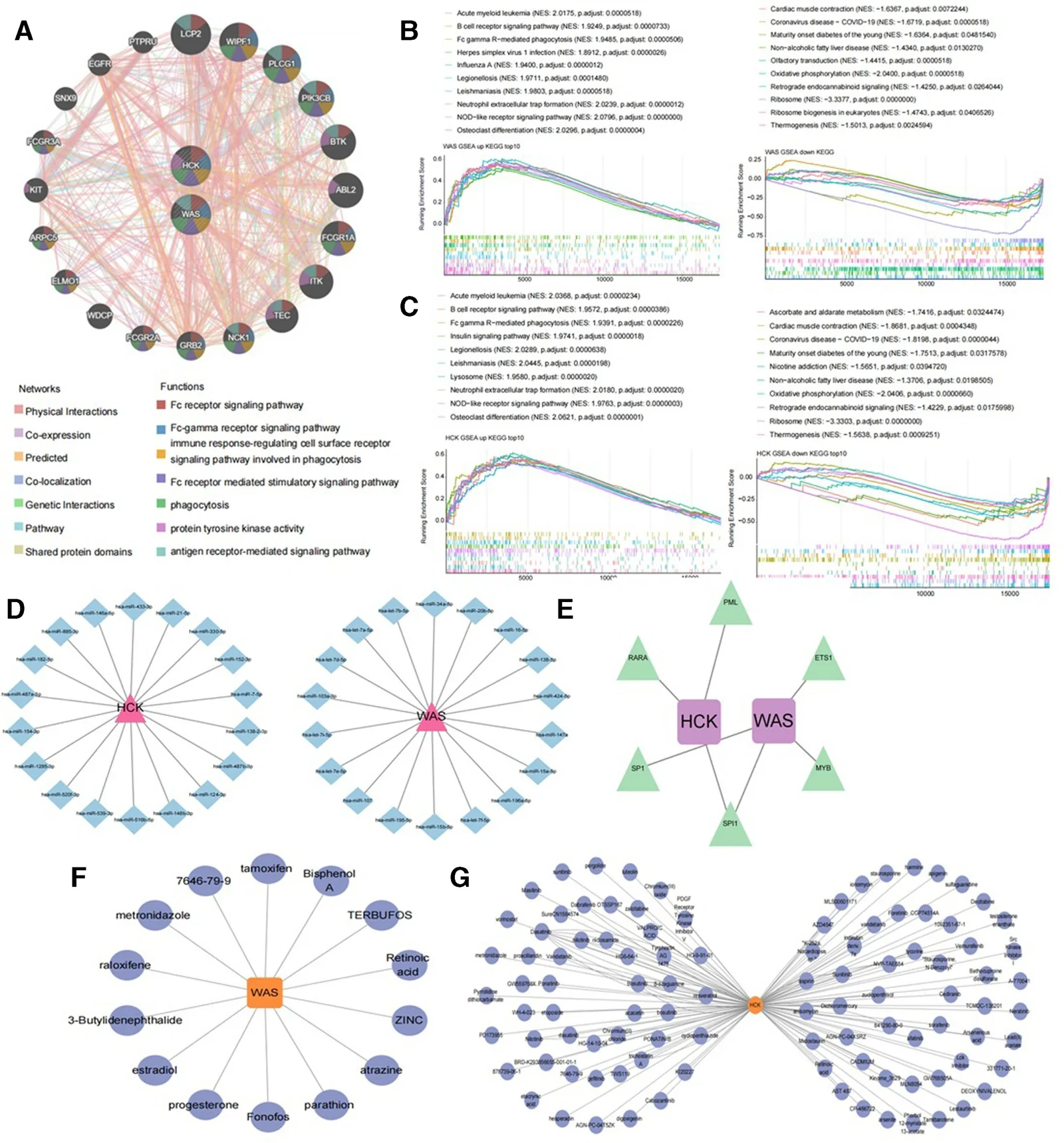
Potential regulatory mechanisms and predicted small-molecule associations of WAS and HCK. (A) Gene–gene interaction (GGI) network generated using GeneMANIA, illustrating functional associations of WAS and HCK with related genes and enriched functional categories. (B) Gene set enrichment analysis (GSEA) of Kyoto Encyclopedia of Genes and Genomes (KEGG) pathways associated with high WAS expression, showing the top upregulated and downregulated pathways. (C) GSEA of KEGG pathways associated with high HCK expression, showing the top upregulated and downregulated pathways. (D) miRNA–mRNA interaction networks showing predicted miRNAs targeting HCK and WAS. (E) Transcription factor (TF)–mRNA regulatory network showing shared and gene-specific TFs regulating HCK and WAS. (F) DSigDB-predicted small-molecule compounds associated with WAS. (G) DSigDB-predicted small-molecule compounds associated with HCK. DSigDB = Drug Signatures Database, GGI = gene–gene interaction, GSEA = gene set enrichment analysis, HCK = hematopoietic cell kinase, KEGG = Kyoto Encyclopedia of Genes and Genomes, TF = transcription factor, WAS = WASP actin nucleation-promoting factor.

miRNA–mRNA network analysis indicated that HCK was associated with 18 miRNAs, including hsa-miR-21-5p and hsa-miR-146a-5p, and WAS with 18 miRNAs, including hsa-miR-34a-5p and hsa-miR-16-5p (Fig. 7D). TF–mRNA analysis suggested that HCK was targeted by SPI1, RARA, and PML, whereas WAS was targeted by SP1, SPI1, ETS1, and MYB, with SPI1 regulating both genes (Fig. 7E).

### 3.8. Small-molecule drug prediction

DSigDB-based prediction identified candidate small molecules associated with WAS (e.g., metronidazole and 3-butylidenephthalide) and HCK (e.g., masitinib and staurosporine; Fig. 7F–G). These results provide hypothesis-generating candidates for experimental prioritization.

### 3.9. Clinical characteristics of the validation cohort

To enhance translational relevance, independent clinical validation was performed using peripheral blood samples from patients with NP (n = 17) and matched healthy controls (n = 17). The baseline demographic and clinical characteristics of NP patients and healthy controls included in the independent clinical validation cohort are summarized in Table 1. No significant differences were observed between groups in age, sex distribution, or body mass index.

**Table 1:** Baseline characteristics of the clinical validation cohort.

Characteristic	NP patients (n = 17)	Healthy controls (n = 17)	*P* value
Age (yrs)	52.4 ± 11.6	50.9 ± 10.8	.699
Sex (male/female)	9/8	8/9	1.000
Body mass index (kg/m^2^)	24.8 ± 3.2	24.1 ± 3.0	.515
Neuropathic pain subtype	Sciatica/DPN/PHN	–	–
Disease duration (mo)	14.0 (8.0–26.0)	–	–

NP = neuropathic pain.

### 3.10. WAS and HCK are upregulated in peripheral blood from NP patients

Quantitative real-time PCR analysis demonstrated significantly higher relative mRNA expression of HCK and WAS in peripheral blood from NP patients (n = 17) compared with healthy controls (n = 17), after normalization to GAPDH using the 2^−ΔΔCt^ method (*P* < .01; Fig. 8A–B).

**Figure 8.**
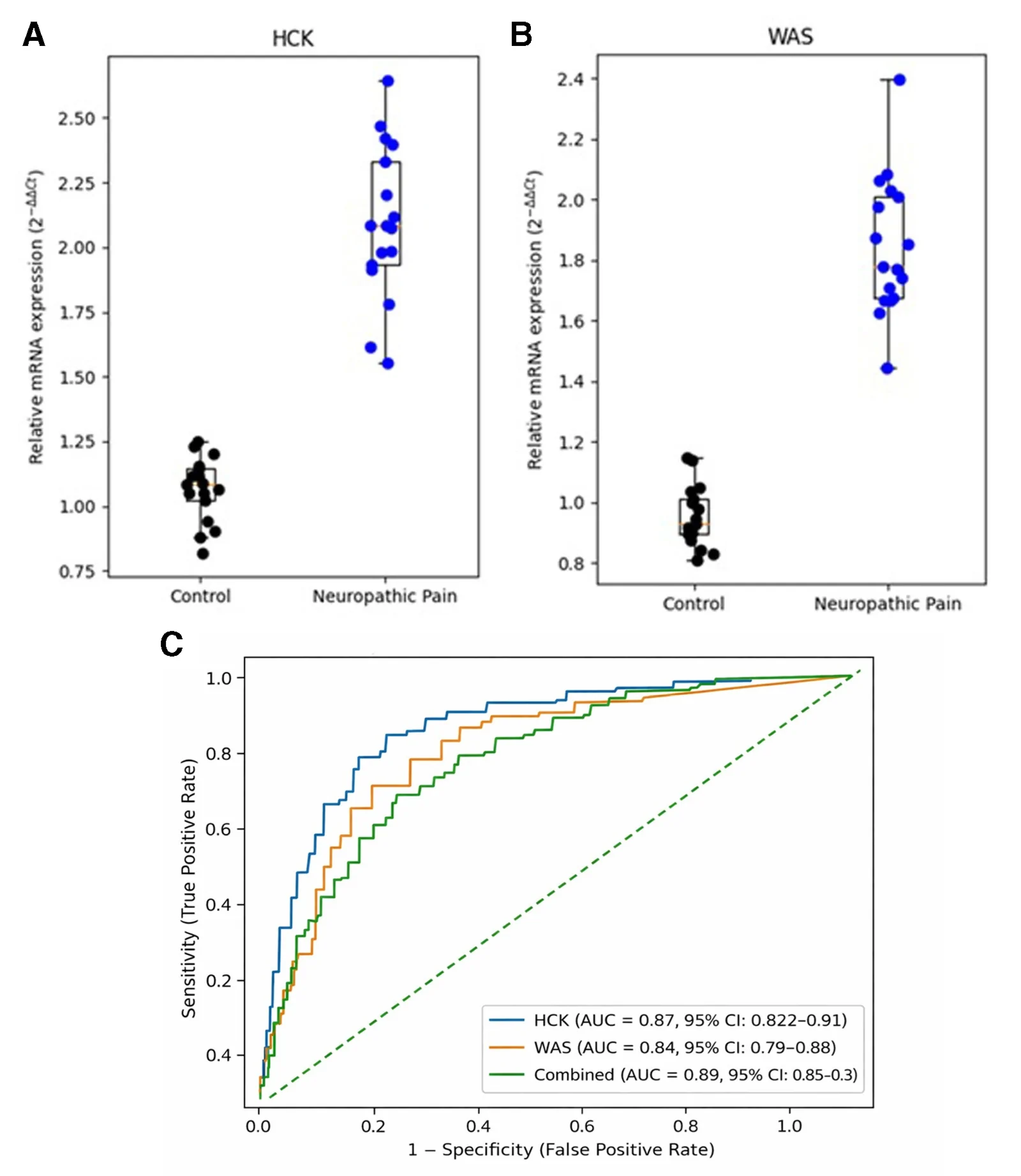
Independent clinical validation of WAS and HCK in peripheral blood. (A) Relative mRNA expression of HCK in peripheral blood samples from neuropathic pain (NP) patients (n = 17) and healthy controls (n = 17), measured by quantitative real-time PCR (qRT-PCR) and normalized to GAPDH using the 2^−^ΔΔCt method. (B) Relative mRNA expression of WAS in NP patients and healthy controls. Individual points represent individual subjects; box plots indicate the median and interquartile range. (C) Receiver operating characteristic (ROC) curves showing the diagnostic performance of HCK (AUC = 0.87, 95% CI: 0.82–0.91), WAS (AUC = 0.84, 95% CI: 0.79–0.88), and the combined WAS + HCK logistic regression model (AUC = 0.89, 95% CI: 0.85–0.93) for distinguishing NP patients from healthy controls. AUC = area under the curve, CI = confidence interval, HCK = hematopoietic cell kinase, WAS = WASP actin nucleation-promoting factor.

### 3.11. Clinical diagnostic performance of WAS and HCK and the combined model

ROC curve analysis in the independent clinical cohort demonstrated good diagnostic performance for HCK (AUC = 0.87, 95% CI: 0.82–0.91) and WAS (AUC = 0.84, 95% CI: 0.79–0.88). A combined diagnostic model integrating WAS and HCK using logistic regression further improved discrimination between NP patients and healthy controls, yielding an AUC of 0.89 (95% CI: 0.85–0.93; Fig. 8C).

## 4. Discussion

NP remains challenging to diagnose and manage owing to its heterogeneous etiologies and the incomplete understanding of underlying neuroimmune mechanisms. In the present study, we integrated lactylation- and palmitoylation-related transcriptomic signals and identified 2 feature genes – HCK (lactylation-related) and WAS (palmitoylation-related) – that were consistently upregulated in NP across multiple GEO discovery and validation datasets. Both genes demonstrated diagnostic potential, supporting the construction of a two-gene nomogram with favorable calibration and decision-curve benefit. Importantly, we further confirmed the upregulation of WAS and HCK in an independent clinical cohort (n = 17 per group) using qRT-PCR, strengthening the translational relevance of these findings. WAS encodes Wiskott–Aldrich syndrome protein (WASp), a key regulator of actin cytoskeleton remodeling and immune synapse formation. WASp facilitates T-cell activation through ARP2/3 complex–mediated actin polymerization and supports downstream transcriptional programs essential for interleukin-2 induction.^[31–35]^ Notably, interleukin-2-related pathways have been associated with antinociceptive effects in experimental models,^[36]^ suggesting that WAS upregulation may reflect immunoregulatory processes linked to pain modulation. HCK, a Src family kinase predominantly expressed in myeloid lineage cells, plays an important role in inflammatory signaling and has been implicated in neuroimmune pathways relevant to migraine and NP.^[37]^ Together, WAS and HCK likely represent immune activation signatures associated with NP, particularly detectable in peripheral blood transcriptomes.

Mechanistically, HCK may provide a link between the observed myeloid-cell signature and neuroinflammation in NP. As a Src-family kinase predominantly expressed in myeloid cells, HCK participates in inflammatory signaling in macrophages and microglia. Notably, HCK has been identified as an upstream regulator of the NLRP3 inflammasome, with HCK activity promoting NLRP3 inflammasome assembly, caspase-1 activation, and IL-1β release.^[38]^ This evidence is particularly relevant to our finding that HCK expression correlated positively with neutrophils, monocytes, and macrophages and was associated with NOD-like receptor signaling. Thus, increased HCK expression may reflect or contribute to a myeloid inflammatory state capable of promoting neuroinflammation and nociceptive sensitization, although this mechanistic relationship requires direct validation in NP models. WAS may contribute through a complementary cytoskeletal mechanism. WASp activates the ARP2/3 complex to promote branched actin polymerization and is recruited to the immunological synapse, where actin remodeling facilitates receptor organization and downstream immune-cell signaling.^[33–35]^ Dysregulation of this process could alter immune-cell activation and intercellular communication, potentially sustaining inflammatory responses relevant to chronic pain. Although a direct role of WAS-mediated immune-synapse remodeling in NP remains to be established, these mechanisms provide a plausible biological basis for the association between WAS expression and immune-cell infiltration observed in our study. Immune infiltration analysis provided convergent evidence supporting this interpretation. Both WAS and HCK expression levels were positively correlated with neutrophils, monocytes, and macrophages – innate immune cell populations known to participate in neuroinflammatory processes and peripheral sensitization. Neutrophils can influence central nervous system barrier integrity and inflammatory signaling,^[39,40]^ while monocytes and macrophages contribute to the release of inflammatory mediators and may directly modulate nociceptor excitability.^[41–44]^ These associations are consistent with the concept that NP is accompanied by systemic immune activation, which can be captured through peripheral blood-based biomarkers. Pathway enrichment analysis further supported the involvement of inflammatory signaling, as both WAS and HCK were associated with the NOD-like receptor signaling pathway. NOD-like receptors play central roles in inflammasome activation and pro-inflammatory cytokine production,^[45]^ and inhibition of NLRP3 inflammasome signaling has been shown to alleviate NP in diabetes-related models.^[46]^ These findings suggest a potential mechanistic link between WAS/HCK dysregulation and inflammasome-mediated neuroinflammation. In addition, regulatory network analyses identified multiple microRNAs with established relevance to pain biology – such as miR-21-5p, miR-146a-5p, miR-34a-5p, and miR-16-5p – as potential upstream regulators of WAS and HCK.^[47–52]^ Transcription factor (TF) analysis further highlighted *SPI1* as a shared regulator of both genes, consistent with its known role in myeloid differentiation and immune transcriptional programming. Although these regulatory relationships remain inferential, they provide a coherent framework for future mechanistic studies.

## 5. Limitations

Several limitations should be acknowledged. First, the discovery transcriptomic datasets were of modest sample size, and relatively permissive DEG thresholds were applied to minimize false negatives, necessitating independent validation. Second, the clinical validation cohort (n = 17 per group) represents an exploratory pilot sample and should be expanded in larger, multicenter studies to refine diagnostic thresholds and assess subtype-specific performance (e.g., diabetic neuropathy vs sciatica). Third, functional experiments were not performed to directly establish causal roles for WAS and HCK or to confirm their lactylation or palmitoylation modification status in NP contexts. Future studies should incorporate mechanistic investigations, including immune cell subset–specific analyses, protein-level validation, and in vivo NP models, to determine whether modulation of these pathways can influence NP pathophysiology.

## 6. Conclusion

This integrative study identified WAS and HCK as lactylation-/palmitoylation-related feature genes associated with NP. Both genes demonstrated diagnostic utility in GEO datasets and were validated in an independent clinical cohort using qRT-PCR. Immune infiltration and enrichment analyses implicated innate immune activation and NOD-like receptor signaling as potential mechanisms. These findings provide candidate blood-based biomarkers and hypothesis-generating therapeutic directions for NP.

## Acknowledgments

The authors thank the clinical staff involved in participant recruitment and sample collection.

## Author Contributions

**Conceptualization:** Honglin Yan, Yanwei Yang.

**Data curation:** Honglin Yan, Lei Li, Jing Li, Junqiang Hao, Yanwei Yang.

**Formal analysis:** Honglin Yan, Lei Li, Jing Li.

**Funding acquisition:** Yanwei Yang.

**Investigation:** Honglin Yan, Lei Li.

**Methodology:** Honglin Yan, Lei Li, Jing Li, Yanwei Yang.

**Project administration:** Junqiang Hao, Yanwei Yang.

**Resources:** Junqiang Hao, Yanwei Yang.

**Software:** Junqiang Hao, Yanwei Yang.

**Supervision:** Yanwei Yang.

**Validation:** Honglin Yan, Yanwei Yang.

**Visualization:** Honglin Yan.

**Writing – original draft:** Honglin Yan, Lei Li, Jing Li.

**Writing – review & editing:** Junqiang Hao, Yanwei Yang.


