# Supplementary material for: Elucidation of key lactylation- and palmitoylation-related genes and their related mechanisms in the development of neuropathic pain
Source: Medicine (Baltimore). 2026 Oct 2;105(40):e50651. doi: 10.1097/MD.0000000000050651 (PMC13633018; doi:10.1097/MD.0000000000050651)
Supplement: Supplementary file 1 [file medi-105-e50651-s001.docx]

**Supplementary Table S1.** Primer sequences used for qRT-PCR analysis

| Gene | Forward primer (5′→3′) | Reverse primer (5′→3′) | Amplicon size (bp)* | Annealing temperature (°C) |
| --- | --- | --- | --- | --- |
| HCK | TGAGGACAACGAGTACACGGCT | CGTCTGACTTGATGGTGAAGGAG | ~120–150 | 60 |
| WAS | CACGAGAACCAGCGACTCTTTG | CCACAATGCTCCTTGGTCCAGT | ~120–150 | 60 |
| GAPDH | GTCTCCTCTGACTTCAACAGCG | ACCACCCTGTTGCTGTAGCCAA | ~130–150 | 60 |
